# American Medical Association

**Published:** 1865-05

**Authors:** 


AMERICAN MEDICAL ASSOCIATION.—The sixth of June, the day appointed for the National Association, is near at hand, and everything appears propitious for a meeting of unusual interest. The State and City authorities have united with the profession of Boston and the reception and entertainment of members will form an attractive feature of the occasion. The social entertainments of these meetings have always been unsurpassed in attractions; nothing better or more satisfactory can be desired in this direction.

The Boston Medical and Surgical *Journal* speaks of the unanimity of the profession of that city and the hearty co-operation of all parties, in making provision for the entertainment of the Association, and extends to the profession and to the Medical Staff of the Army and Navy a polite and cordial invitation to be present and participate in the proceedings of the Association. We hope there will be a full attendance, and that the coming meeting will be productive of substantial good to the profession.

